# Long-chain surface-modified red-emitting carbon dots as fluorescent additives for 3D printing vat-photopolymerization†

**DOI:** 10.1039/d4na00617h

**Published:** 2024-11-26

**Authors:** Simone Maturi, Andrea Baschieri, Erica Locatelli, Martina Buccioli, Mauro Comes Franchini, Letizia Sambri

**Affiliations:** a Department of Industrial Chemistry “Toso Montanari”, University of Bologna Via Piero Gobetti 85 40129 Bologna Italy letizia.sambri@unibo.it; b Institute for Organic Synthesis and Photoreactivity (ISOF), National Research Council of Italy (CNR) Via Piero Gobetti 101 40129 Bologna Italy

## Abstract

Carbon dots have recently attracted tremendous scientific attention thanks to their enhanced luminescence properties, photostability and low toxicity. In particular, red-emitting carbon dots (RCDs) are assuming increasing importance in biomedical applications, such as bio-imaging and phototherapy. At the same time, the possibility to create functional and complex objects by means of vat-photopolymerization-based three-dimensional (3D) printing techniques is continuously growing. This work describes the synthesis of long-chain surface-modified red emitting carbon dots, L-RCDs by esterification of RCDs, obtained from green reagents with a new solvothermal synthesis, and their employment as fluorescent additives in two formulations of photopolymerizable resins. The printing process proceeded smoothly in all cases, and red-emitting objects with different mechanical properties have been successfully obtained.

## Abstract 2

Red-emitting carbon dots from renewable materials can be used as fluorescent additives in 3D-printing vat-photopolymerization.

## Graphical abstract

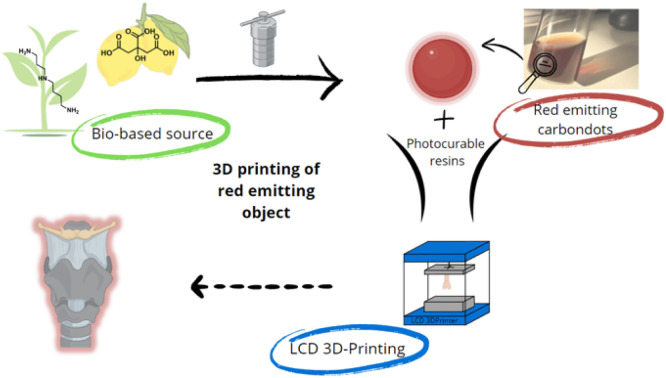

## Introduction

Initially discovered in 2004,^1^ carbon dots (CDs) are currently considered rising stars in the universe of carbon nanoparticles owing to their excellent luminescence properties, low toxicity, high biocompatibility and photostability, and to the possibility of surface functionalization.^2–4^

Detailed descriptions, classifications and synthetic methods recently appeared in various reviews.^5,6^ In particular, CDs synthesized from small molecules through a bottom-up approach exhibit high versatility in terms of a huge variety of low cost and green starting materials, cheap and simple synthetic procedures and tuneability of the colour of the emitted light.^7–9^

Currently, CDs find application in a wide variety of fields, from sensing to catalysis and from energy to biomedical applications,^8,10–12^ among others. Specifically, even though most reported methodologies afford blue or green emitting carbon dots, during recent years tremendous efforts have been devoted to the development of synthetic procedures providing carbon dots exhibiting good optical properties in therapeutic windows, *i.e.* absorption and emission in the red to NIR region; in fact, one of the main advantages of red emitting carbon dots is that their emission can penetrate deep into biological tissue without any interference from the tissue's fluorescence, so that they can be exploited for efficient biomedical applications, such as bioimaging and even phototherapy.^13–15^

Notably, owing to the increasing demand for new smart materials, CDs display great potentiality to be used as additives at a low percentage for the development of advanced functional materials for application in the most innovative production.

In this framework, 3D-printing is one of the most recent techniques which has been undergoing fast and exponential growth in the last few years, since its approach is highly sustainable being fast, cheap, and versatile, allowing the use of 3D design software to create a real physical object potentially with no limits in terms of geometry, details, dimensions, and accuracy.^16–18^

3D printing technologies based on the vat-photopolymerization of appropriate resins, such as LCD (Liquid Crystal Display), SLA (stereolithography) and DLP (Digital Light Processing), offer some advantages over the extrusion approach, such as a more accurate resolution and speed.^19–22^ Furthermore, the opportunity to design and vary photocurable monomers leads to massive chemical research opportunities because each component can promote substantial differences in the mechanical properties of the final 3D printed object. Moreover, in principle, specific additives with given functions can be incorporated into the resins with the aim to produce functional objects.

Therefore, we focused on the fluorescence properties of carbon dots and on the challenge to transfer them to three-dimensional printed objects. Their use as additives in 3D printing is in fact still unexplored; few examples appeared in the literature about the use of CDs in 3D printing applications, particularly referring to materials for filament extrusion techniques to obtain fluorescent objects.^23–25^ Only very recently CDs have been reported in 3D-printing using resins exploiting their optical characteristics surprisingly acting as photoinitiators,^26,27^ and to the best of our knowledge, this is the only reported case with CDs in vat-photopolymerization.

From our point of view, there is a substantial lack of investigation on the surface functionalization of carbon nanodots and this could be the reason for the few studies related to the incorporation of these nanoparticles into the common lipophilic mixtures of resins for vat-photopolymerization.

Indeed, their incorporation as surface esterified luminescent additives in photocurable resins, without substantial modification of the technological performances in the final object, still lacks a deep discussion.

Herein, we report the synthesis of long-chain surface-functionalized red emitting carbon dots and their use as additives to resins with different mechanical properties to demonstrate the possibility to print red-emitting 3D objects for potential applications in various fields, from decorative manufacturing to implantable and biomedical tool production, without any significant loss of mechanical properties.

## Experimental

### Materials and methods

Citric acid 99%, dodecanol, dimethylformamide (DMF), glycerol propoxylate triacrylate (GPT), glycerol dimethacrylate (GDMA) and ethyl (2,4,6-trimethylbenzoyl) phenylphosphinate (Et-APO) have been purchased from Sigma-Aldrich; spermidine trichloride 99+% has been provided by Acros Organics; formamide 99% has been provided by VWR; 2,6-di-*tert*-butyl-4-methylphenol (BHT), phenoxyethylacrylate (PEA) and 1,6-hexanediol diacrylate (HDDA) have been purchased from TCI Chemicals; 1,2-cyclohexane dicarboxylic acid and diisononyl ester (DINCH) have been provided by BASF. Sigma-Aldrich provided acetone and ethanol in analytical grade while isopropanol and dichloromethane in GC grade (≥99.8%). All the reagents and solvents have been used without any further purification.

### Synthesis and purification of RCDs

Citric acid (300 mg, 1.56 mmol) was dissolved in formamide (20 mL) under continuous stirring at room temperature in a stainless-steel autoclave. After complete solubilization, spermidine (1.1 g, 7.57 mmol) was added. Then the autoclave was sealed, covered with aluminium foil, and placed on a magnetic stirrer plate for heating at 190 °C for 4 h under stirring (400–600 rpm). Once the time was over, the heating was turned off and the system was allowed to reach the room temperature overnight. To purify the synthesized RCDs, the obtained dark red solution was filtered with a 0.22 μm Sterivex filter and dripped in 200 mL of acetone. The suspension was centrifuged in Falcon tubes at 9000 rpm for 20 min, then the surfactant was discarded and the solid residue was washed with a solution EtOH : acetone 1 : 1 and centrifuged. Again, the surfactant was discarded, and the solid was suspended in DCM and decanted in a pear-shaped flask for about 4 h. The solvent was then removed and the solid was fully dried using a rotary evaporator to give 220 mg (73% yield in mass in relation to the mass of starting citric acid) of red-brownish RCDs that were then stored in a capped vial.

### Synthesis and purification of L-RCDs

RCDs (50 mg) were dissolved in DMF (10 mL) in a 50 mL round bottom flask, and then dodecanol (20 mL) and sulfuric acid (100 μL) were added. The mixture was refluxed at 155 °C under nitrogen for 90 min and then allowed to reach room temperature. The long-chain surface-modified RCDs (L-RCDs) were purified by dripping the obtained solution in 150 mL of acetone followed by the centrifugation of the resulting suspension at 9000 rpm for 20 min. The surfactant solvent was discarded and the solid was suspended in DCM and transferred into a pear-shaped flask to decant for about 4 h. After the solvent removal, the brownish solid was dried using a rotary evaporator to give 30 mg of L-RCDs (60% yields w/w) that were then stored in a capped vial.

### Formulation of resins

The two selected resins were formulated with different acrylates and some common components. In detail, the first one, Resin-R, optimized to exhibit hardness and rigidity, contained glycerol propoxylate triacrylate (GPT) and glycerol dimethacrylate (GDMA) in ratio 1 : 1; the second one, Resin-F, with flexible characteristics, was formulated by adding phenoxyethylacrylate (PEA) and 1,6-hexanediol diacrylate (HDDA) in ratio 7 : 3.

The following reagents were added in the same weight proportions to both the resins: the photoinitiator ethyl (2,4,6-trimethylbenzoyl) phenylphosphinate (Et-APO) (0.5%), the terminator 2,6-di-*tert*-butyl-4-methylphenol (BHT) (0.5%), and the plasticizer 1,2-cyclohexane dicarboxylic acid diisononyl ester (DINCH) in 7% in weight.

To the starting resins, the carbon nanoparticles were then added in four different percentages 0.01%, 0.05%, 0.1% and 0.2% in weight.

All the components have been weighed in a jar of the specific resin mixer in order to homogenize all the components. To make the dispersion of the nanoparticles easier the liquid resin was ultrasonicated (40 kHz) for five min.

### LCD 3D printing

The dog-bone 3D model has been downloaded from an open-source website and subsequently the digital model has been sliced with ChiTu Box software provided by the printers' company. A Phrozen Sonic Mini 4K LCD 3D printer has been used for the 3D printing of all specimens; this printer was equipped with an LCD screen (405 nm ParaLED Matrix 2.0, 40 W system power). This printer offered an XY resolution of 35 μm and a layer thickness in the range of 0.01–0.30 mm. The total printing area was 13.4 × 7.5 × 13 cm with a maximum vertical printing speed of 80 mm per hour.

Several parameters have been optimized to obtain the best resolution and the structural integrity of the final printed specimen. The two different tested resins required their own specific optimization in terms of exposure time for each layer which turned out to be very different from resin to resin. The rigid Resin-R required an exposure time of 50 s, and the flexible Resin-F required 25 s.

Based on the time for each layer and the height of the specimen for tensile tests the printing time took from 15 min for the flexible Resin-F to 40 min for the rigid Resin-R. After the printing of the 3D model, the specimens were removed from the printing plate and washed carefully with 2-propanol to remove all the non-printed liquid resin. The specimens were then post cured for 1 minute each side in a UV curing oven (Sharebot CURE, Nibionno, Italy, *λ* = 375–470 nm, 120 W) and then left exposed to environmental light and air for one week before the tensile tests.

### Characterization

ATR-FTIR analysis has been performed using a PerkinElmer Spectrum 2000 FT-IR spectrophotometer. Samples for the transmission electron microscopy (TEM) analysis were prepared by adding a drop of nanoparticle suspension on a copper TEM grid. The conventional transmission electron microscopy (TEM) imaging was performed by applying a Cs-aberration-corrected probe (JEM-ARM 200CF; JEOL, Tokyo, Japan) using a cold field emission source operated at 200 keV, with a spatial resolution in STEM imaging mode of 0.1 nm. *Z*-potential measurements have been performed using a Malvern Instruments – Zetasizer Nano ZS and placing the samples in disposable folded capillary cells in distilled water solution. For room-temperature absorption and emission experiments in solution, carbon dots were dissolved in bi-distilled water and placed in fluorimetric Suprasil quartz cuvettes (10.00 mm), while for solid 3D-printed resins, samples were placed between two quartz slides. Absorption spectra have been recorded with a PerkinElmer Lambda 950 spectrophotometer. Reflectance spectra were acquired with the spectrophotometer described above, equipped with a 100 mm integrating sphere. The uncorrected emission spectra were obtained with an Edinburgh Instruments FLS920 spectrometer equipped with a Peltier-cooled Hamamatsu R928 photomultiplier tube (PMT, spectral window: 185–850 nm). An Osram XBO xenon arc lamp (450 W) was used as the excitation light source. Emission spectra for solid samples were collected in the front-face mode. The corrected spectra were acquired by means of a calibration curve, obtained using an Ocean Optics deuterium–halogen calibrated lamp (DH-3plus-CAL-EXT). The photoluminescence quantum yields (PLQYs) in solution were obtained from the corrected spectra on a wavelength scale (nm) and measured according to the approach described by Demas and Crosby,^28^ using an air equilibrated ethanol solution of rhodamine 6G as a reference (PLQY = 0.94).^29^ The emission lifetimes (*τ*) were measured through the time-correlated single photon counting (TCSPC) technique using a HORIBA Jobin Yvon IBH FluoroHub controlling a spectrometer equipped with a pulsed NanoLED (*λ*_exc_ = 465 nm) as the excitation source and a red-sensitive Hamamatsu R-323701 PMT (185–850 nm) as the detector. The analysis of the luminescence decay profiles was accomplished with the DAS6 Decay Analysis Software provided by the manufacturer, and the quality of the fit is assessed with the *χ*^2^ value close to unity and with the residuals regularly distributed along the time axis. Experimental uncertainties were estimated to be ±8% for *τ* determinations, ±10% for PLQYs, and ±2 and ±5 nm for absorption and emission peaks, respectively.

A Remet TC10 universal testing machine was used to perform all the tensile tests. The instrument was equipped with a 1 kN cell with crosshead separation speed of 1 mm min^−1^ for the rigid Resin-R and for the flexible Resin-F.

## Results and discussion

### Synthesis of RCDs and L-RCDs

Beside the use of aromatic or highly conjugated molecules as precursors for the synthesis of red emitting carbon dots, doping with heteroatoms from nitrogen rich starting materials and solvents is a useful strategy to red-shift their emission.^30^

**Scheme 1:**
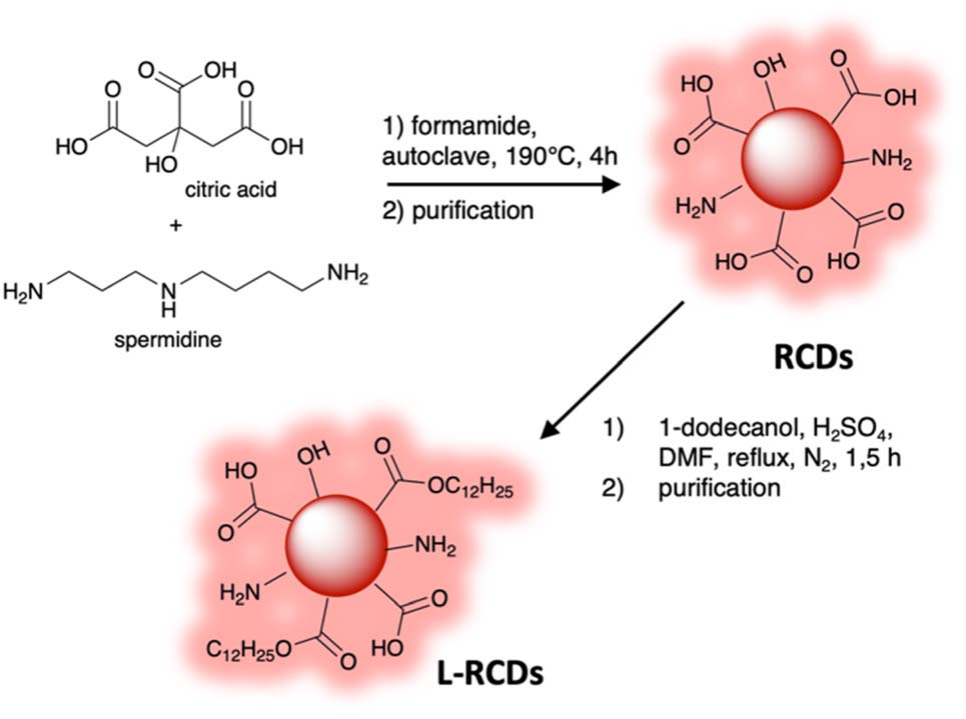
Synthetic route to RCDs and L-RCDs.

Herein we synthesized red emitting carbon dots RCDs adopting a bottom-up approach and, analogously to previous reported procedures,^5,15^ we used a solvothermal method exploiting renewable and bio-based reagents, *i.e.* citric acid and spermidine,^31,32^ by heating the mixture in an autoclave at 190 °C for 4 h in formamide.^33,34^ Then a convenient purification procedure that avoids column chromatography was set up: at the end of the reaction the cooled mixture was filtered with a Sterivex filter and dripped into acetone to precipitate the nanoparticles. The obtained suspension was centrifuged; then, after removal of the supernatant, the solid was washed with a fresh solution of acetone/EtOH. The obtained dark-red carbon dots were suspended in DCM, then recovered after decanting and finally characterized (Scheme 1).

The reaction conditions, the use of N-containing reagents and a careful purification procedure let us obtain carbon nanoparticles showing remarkable red-emission and containing various polar functional groups on their surface, as detailed below, that permitted further functionalization.

In fact, since the final goal of our research was to employ red-emitting carbon dots as additives for photopolymerizable resins, the polarity of the nanoparticles could influence their dispersion in polymeric matrices, namely acrylate derivatives.

We therefore modified their surface by esterification of some of the carboxylic groups with a long chain alcohol, specifically 1-dodecanol, to decrease their hydrophilicity and to increase their affinity and dispersion within the resins.

Some of the obtained RCDs were therefore refluxed in DMF with a large excess of 1-dodecanol under acidic catalysis to perform Fisher condensation leading to the corresponding ester derivatives L-RCDs.^35^ The success of the reaction was immediately clear observing the change in solubility of the obtained nanoparticles: contrary to the starting carbon dots, they were completely soluble in the acetone/EtOH = 1 : 1 mixture used for the purification of RCDs, so we brought some changes in that step and L-RCDs were purified by precipitation in acetone, centrifugation and decanting in DCM, giving dark-red nanoparticles. However, as reported in the following characterization description, probably due to steric concerns, only some of the carboxylic groups present on the carbon dots' surface underwent esterification allowing the obtained L-RCDs to be still soluble in water.

### Characterization of RCDs and L-RCDs

After the purification, the nanoparticles were characterized with different techniques, that confirmed the presence of various functional groups on their surface, such as –COOH, –OH and –NH_2_, together with the aromatic core responsible for their optical properties.

**Fig. 1:**
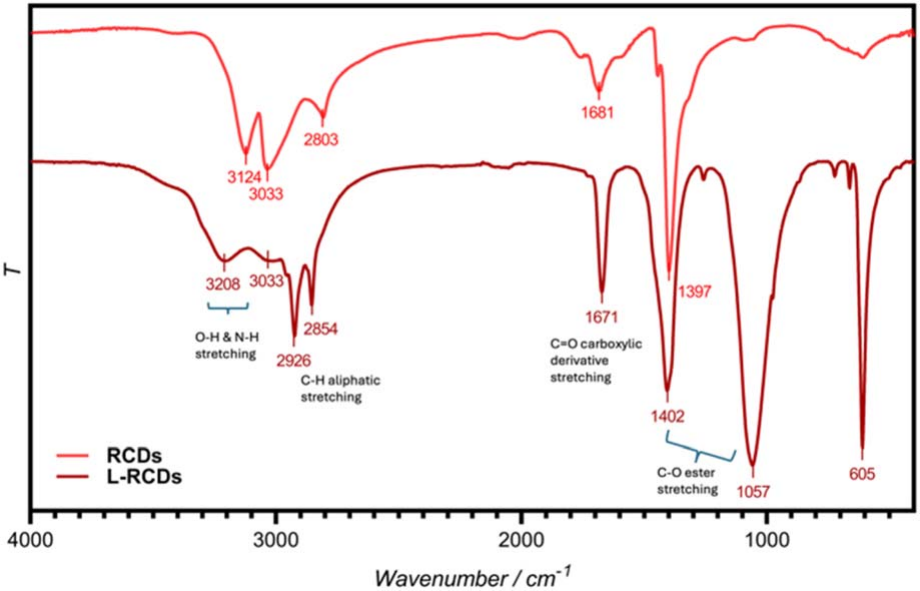
FT-IR spectra of RCDs and L-RCDs in water solution.

First, the recognition of functional groups in the synthesized carbon dots was obtained from the FTIR spectra (Fig. 1). The pristine RCDs and the ester-functionalized L-RCDs presented pronounced bands in the 3200–3000 cm^−1^ range that can be ascribed to the stretching of N–H and O–H groups. The peaks at 1700–1600 cm^−1^ were due to stretching frequency of C=O of carboxylic derivatives on the surface (acids, amides and then esters). The L-RCD spectrum is distinguished from the previous one by the presence of some additional bands at 2926 and 2854 cm^−1^_,_ corresponding to the stretching vibration of –CH_2_ aliphatic groups, and at 1057 cm^−1^, that, together with the one at 1402 cm^−1^ corresponded to the C–O stretching in esters, thus indicating the success of the esterification that occurred with the formation of covalent bonds.

**Fig. 2:**
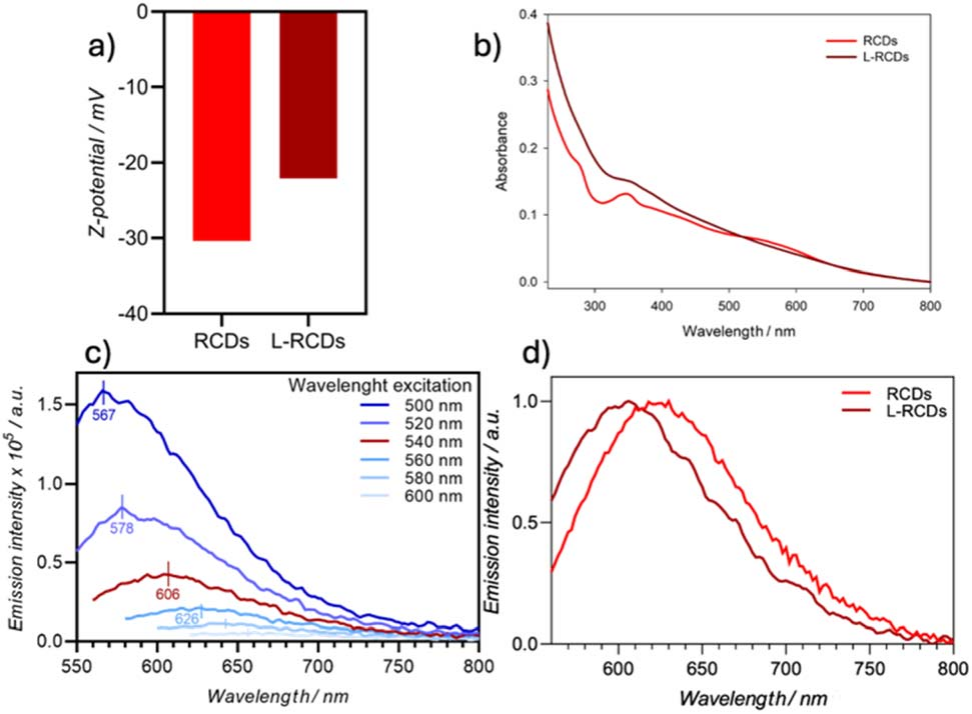
(a) *Z*-potential values of RCDs and L-RCDs; (b) absorption spectra of RCDs (0.102 mg mL^−1^) and L-RCDs (1.056 mg mL^−1^) in room-temperature water solutions; (c) emission spectra of L-RCDs in H_2_O at 298 K upon excitation in the range of 500–600 nm; (d) normalized emission spectra of RCDs and L-RCDs in H_2_O (*λ*_ex_ 540 nm).

Then, the zeta-potentials of the synthesized fluorescent RCDs and ester-functionalized L-RCDs were measured obtaining their respective surface charges. The presence of carboxylic groups that are deprotonated at neutral pH (COO^−^) was confirmed by the negative surface charge (−30.4 mV) of RCDs (Fig. 2a). Accordingly, the zeta-potential of L-RCDs was found to be less negative, *i.e.* −22.1 mV, indicating that some of the –COOH surface groups transformed into neutral –COOR groups (R = C_12_H_25_) with a consequent decrease in the negative surface charge.

The dimensional characterization, provided by TEM analysis, showed that the synthesized L-RCDs were uniformly dispersed nanoparticles with a circular shape and an average diameter of about 5.35 nm (SD = 1.03), similarly to the pristine RCDs (Fig. S1†).

The optical properties of L-RCDs were then evaluated through UV-vis absorption and photoluminescence emission in H_2_O (Fig. 2b–d) and compared to those of RCDs. The UV-vis absorption spectra of both L-RCDs and RCDs displayed a strong absorption throughout the visible region. They showed a maximum peak in the UV region at 370 and 350 nm respectively, probably due to the π–π* transition of the graphitic sp^2^ domain and broadened absorbance in the 500–600 nm range with a shoulder peak at around 560 nm for RCDs, ascribed to the n → π* transition of aromatic and heteroaromatic systems (Fig. 2b). The photoluminescence of L-RCDs in H_2_O was measured upon excitation in the range between 500 and 600 nm showing that, like reported in the literature for other kinds of carbon dots,^36^ the emission is dependent on the excitation wavelength, and it occurs up to the red region (Fig. 2c). Upon excitation at 540 nm, a maximum emission peak is obtained at 606 nm that seems to be a good compromise between the emission intensity and its energy. As expected, L-RCDs are fluorescent, with a lifetime of 6.4 ns and a PLQY around 1.2% (Table 1, entry1).

**Table 1:** Luminescence properties of carbon dots L-RCDs and RCDs in water solution at 298 K

Entry	Sample	*λ*_abs_ [nm]	*λ*_em_ a [nm]	PLQY^b^ [%]	*τ* c [ns]
1	L-RCDs	370	606	1.2	6.4
2	RCDs	350, 560	625	1.9	6.8

a *λ* _exc_ = 540 nm.

b *λ* _exc_ = 480 nm.

c *λ* _exc_ = 465 nm.

The optical properties of the carbon dots before the esterification did not show appreciable differences (Table 1, entry 2 and Fig. S2†), if not a slight red shift of the *λ*_max_, as can be appreciated by comparing the normalized emissions of the carbon dot solutions upon excitation at 540 nm (Fig. 2d) and that can be ascribed to the changes in the functional groups on the nanoparticles' surface.

Notably, it is important to mention that, since it was not possible to determine the molecular weight of the carbon dots, we could measure their amounts only by weight and not by moles in the printing experiments discussed below. However, it appeared reasonable that since the carbon dots' absorption and emission properties could be ascribed only to the graphitic core of the nanoparticles and not to the alkyl chains, weighing the same amounts of RCDs and L-RCDs resulted in a higher amount of absorbing and emitting centres in the former than in the latter ones. Therefore, the absorbance spectra could be a tool to roughly estimate the presence of absorbing centres; in fact, to obtain a similar absorbance intensity, we had to use around ten times, in weight, of L-RCDs with respect to RCDs.

### Resin formulation and vat photopolymerization

**Fig. 3:**
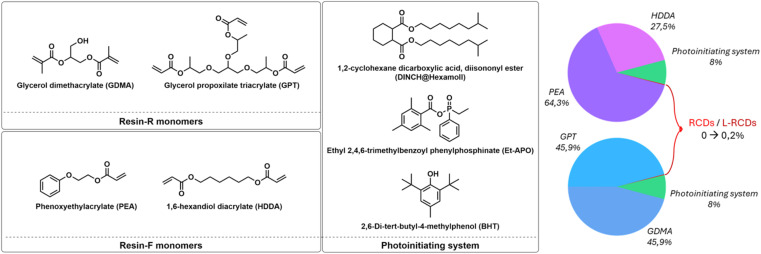
Structures of the components for the resins' formulations.

Depending on the final use, a 3D-printed object could require different levels of rigidity and flexibility. We therefore set up two different resin formulations that, after vat-photopolymerization, afforded objects with different mechanical features. The components we selected for this study were (*meta*)acrylate esters of alcohols and polyols generally employed for this kind of 3D printing^37,38^ (Fig. 3): depending on the desired characteristics of the final objects, mono- or polyfunctionalized, linear or branched monomers have been employed. Then, after the set-up of the resins' formulations, increasing amounts of carbon dots were added to the mixtures to evaluate the best conditions of concentration and characteristics of nanoparticles to obtain reliable red-emitting 3D samples. Remarkably, in all cases, the printing process occurred smoothly without any interference of the photo-active carbon dots with the photopolymerization reaction, and we were able to obtain self-sustainable objects. Finally, the effect of the addition of the carbon dots on the optical and mechanical characteristics of the obtained objects was evaluated.

The first Resin-R, the more rigid one, was formulated using two different glycerols with various acrylic functional groups that, under light emission, highly polymerized to give tough objects. In detail, we mixed in an equal mass ratio the branched glycerol propoxylate trimethacrylate (GPT), containing three different methacrylic units, and glycerol dimethacrylate (GDMA), that carries two terminal methacrylic units (Fig. 3). To obtain flexible and bendable objects, the second Resin-F was formulated with a 1 : 1 mass ratio of unbranched monomers, phenoxyethylacrylate (PEA) and 1,6-hexanediol diacrylate (HDDA), as depicted in Fig. 3.

Both mixtures were added with the same amounts of other components fundamental for the printability of the resin, *i.e.* the photoinitiator ethyl (2,4,6-trimethylbenzoyl) phenyl-phosphinate (Et-APO, 0.5% wt), the radical terminator 2,6-di-*tert*-butyl-4-methylphenol (BHT, 0.5% wt) and the plasticizer, the 1,2-cyclohexane dicarboxylic acid diisononyl ester (DINCH, 7% wt), Fig. 3. Notably, to try to avoid interference in the emission of the final printed objects containing light emitting carbon dots, we excluded from all the formulations any use of photo-absorbers, such as, for instance, isopropyl-thioxanthone (THX), without observing any problem in the printing process and/or in the resolution of the final objects.

**Fig. 4:**
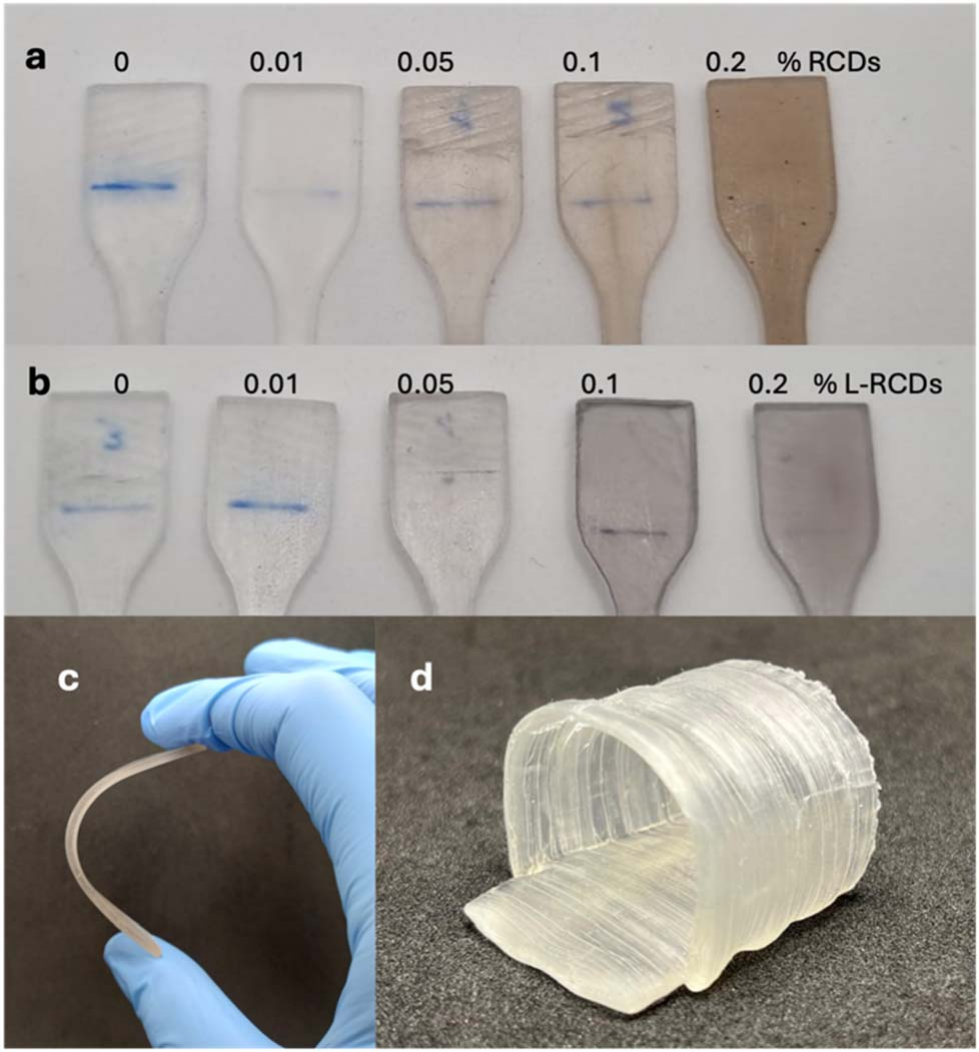
Samples of dog-bones (after tensile tests) printed with Resin-F containing increasing amounts of RCDs (a) and L-RCDs, (b) (0, 0.01, 0.05, 0.1 and 0.2% w/w); (c) flexibility of F@L-RCDs-0.2%; (d) a trachea printed with Resin-F@L-RCDs-0.2%.

The printing and curing parameters were optimized for each resin, and some reference dog-bones were printed. Therefore, we added to the two pristine resins increasing amounts (from 0.01% to 0.2% w/w) of the long-chain surface-modified carbon nanoparticles L-RCDs, and, after homogenization, four samples for each resin containing different amounts of carbon dots were printed to determine the best concentration for each kind of material. The same procedure was repeated using the starting RCDs for comparison. The printing process occurred smoothly with the addition of both nanoparticles, using the same printing parameters as those for the pristine resins, indicating that the amount of the added carbon dots didn't influence the kinetics of the polymerization. In fact, the main goal of this work was to obtain homogeneous resins containing carbon dots that could undergo vat photo-polymerization affording 3D objects that emit red light without losing mechanical properties with respect to those of samples printed with pristine resins. Some pictures of the printed samples with Resin-F are shown in Fig. 4. As can be noted in Fig. 4a, the formulations with 0.2% in weight of not-functionalized RCDs resulted too concentrated and, in fact, aggregations appeared in the printed samples for both resins; on the contrary, homogeneous samples were obtained with all concentrations of long-chain surface-modified carbon dots L-RCDs (Fig. 4b). However, since the nanoparticles' concentrations can be reported only in mass and not in moles, as discussed above, the comparison of the different concentrations is reliable in terms of optical properties only within the same additive species.

All the printed objects were characterized, as detailed below, and, using the flexible Resin-F, an example of a trachea was printed (Fig. 4d) as a proof of concept of possible future applications in implantable objects.

### Characterization of the 3D-printed resins

The 3D-printed materials were spectroscopically and mechanically characterized to determine the effect of the addition of carbon nanoparticles to the pristine formulations.

ATR-FTIR spectroscopy was useful to confirm the effective polymerization of the resin. From the comparison of the spectra of the 3D printed objects and the two uncured resins (Fig. S3 and S4†) we observed that the absorptions typical of unsaturated monomers, *i.e.*, the stretching of the C=C group at 1640 cm^−1^, and the ones attributed to C=C–H deformations at around 940 and 810 cm^−1^, presented a strong reduction in intensity after the printing process, indicating the formation of a network of saturated C–C bonds thanks to the reaction of the unsaturated groups. In addition, in all printed resins the C–H stretching of the alkyl chains was still present in the 2980–2860 cm^−1^ region, together with the C=O stretching at around 1720–1730 cm^−1^. When the starting monomer PEA containing an aromatic group was used (Resin-F), aromatic C=C bending bands were also present in the range of 1480–1600 cm^−1^ together with aromatic C–H bending at 756 and 696 cm^−1^. However, comparing the resins containing carbon nanoparticles, it was not possible to observe any difference from the pristine ones, owing to the relatively small amount of carbon dots added to the resins (Fig. S3 and S4†).

The UV-vis absorption properties of the printed samples were evaluated through reflectance measurements and compared to the reflectance of the carbon dots (Fig. S5–S7†). As expected, in all cases, the reflectance diminished in the visible range with the addition to the resins of both kinds of carbon nanoparticles. At the same concentration (0.1% w/w), the absorption was less pronounced for L-RCDs than for RCDs since they contain fewer chromophoric groups, as confirmed from the spectra of the solid starting nanoparticles, RCDs and L-RCDs.

Then, we measured the emission properties of the samples to check if the red emission of carbon dots can be transferred to the printed objects. First, the pristine resins were analysed: both resins exhibited an emission centred around 450 nm (Fig. S8†), and the emission continued up to 600 nm; therefore possible interference with the emission of the carbon dots subsequently added should be checked.

Since the carbon dots in solution exhibited the highest emission intensity by exciting at 500 nm, we used this excitation wavelength to compare the relative emission of the printed samples with different concentrations in carbon dots so that we could better appreciate the differences.

**Fig. 5:**
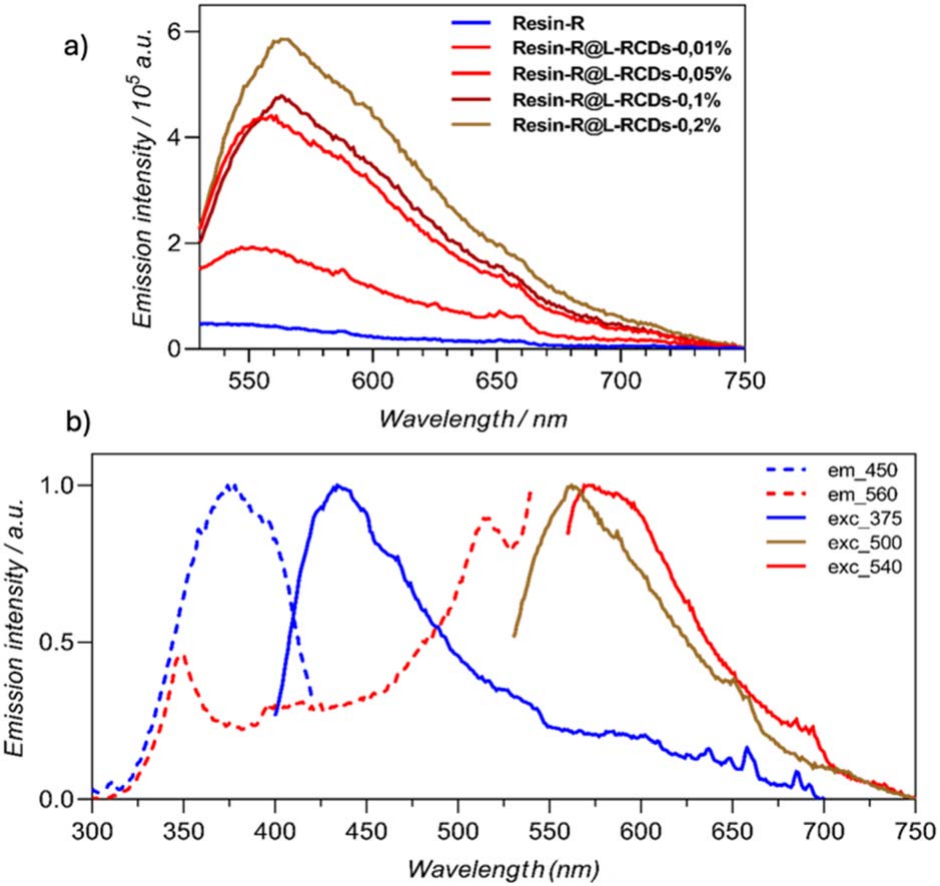
(a) Screening of the emission of Resin-R containing increasing amounts of L-RCDs (*λ*_exc_ = 500 nm); (b) normalized excitation (dashed) and emission (solid) spectra of printed Resin-R@L-RCDs-0.2% at different wavelengths.

Concerning Resin-R, the sample containing the highest amounts of L-RCDs (0.2% in weight) showed the highest emission, as reported in Fig. 5a. Emission in the red region can be recorded upon exciting the samples at 540 nm, as could be observed in the normalized graphic (Fig. 5b), that reported the emissions and the excitations of a printed sample of Resin-R@L-RCDs-0.2% at different wavelengths. The maximum of the emission (*λ*_max_ = 580 nm, Table 2, entry 1) resulted in a blue-shift with respect to the emission of L-RCDs in water solution; however, an appreciable emission could be detected up to 720 nm. Notably, upon excitation at 375 nm, the observed emission (*λ*_max_ = 440 nm) could be ascribed to the polymeric matrix (see also Fig. S8†). In contrast, RCDs could be added to Resin-R only up to 0.1% w/w since a severe decrease in fluorescence was observed with higher amounts (Fig. S9†), although the emission properties appeared similar to those recorded for Resin-R@L-RCDs (Table 2, entry 2 and Fig. S9 and S10†).

**Table 2:** Emission maxima (*λ*_ex_ = 540 nm) of the different printed objects with Resin-R and Resin-F containing the best additive concentration

Entry	Sample	*λ*_em_ [nm]	Conc. (%w/w)
1	Resin-R@L-RCDs	580	0.2
2	Resin-R@RCDs	590	0.1
3	Resin-F@L-RCDs	575	0.2
4	Resin-F@RCDs	575	0.1

Similar results were recorded with the flexible Resin-F: the highest emissions were observed with a concentration of 0.2% and 0.1% in weight of L-RCDs and RCDs respectively (Fig. S11–S14†), and the maxima were both recorded at 575 nm, when excited at 540 nm. It has to be said that pristine Resin-F, however, emitted itself over 550 nm in a detectable amount, so the emission of the carbon dots is slightly disturbed.

Finally, the mechanical properties of the printed resins have been determined through destructive mechanical tensile tests on 3D-printed dog-bone specimens to evaluate the effect of the nanoparticles on the mechanical properties of the printed materials, as reported in Table 3 and in Fig. S15.† Concerning Resin-R, the addition of a 0.2% of L-RCDs slightly decreased the Young's modulus with respect to the pristine resin (−7.1%, Table 3, entries 2 and 1), whereas with only 0.1% of uncoated RCDs the decrease in the Young's modulus was almost double, −12.9% (Table 3, entries 3 and 1). The elongation at break has shown an impressive advantage of using L-RCDs; in fact, starting from a value of 4.2 ± 0.7 for pristine Resin-R we recorded 2.2 ± 0.5 with the addition of 0.1% RCDs (−47.6%) to be compared with 3.9 ± 0.4 (−7.1%) using 0.2% L-RCDs (see Table 3, entries 1–3).

**Table 3:** Selected tensile test results on resins containing L-RCDs and RCDs and pristine resins

Entry	Sample	Young's modulus (Mpa)	Elongation at break %
1	Resin-R	1027 ± 32	4.2 ± 0.7
2	Resin-R@L-RCDs-0.2%	954 ± 56	3.9 ± 0.4
3	Resin-R@RCDs-0.1%	895 ± 41	2.2 ± 0.5
4	Resin-F	12.4 ± 0.7	17.1 ± 0.8
5	Resin-F@L-RCDs-0.2%	11.0 ± 1	16.5 ± 0.8
6	Resin-F@RCDs-0.1%	11.5 ± 0.3	14.2 ± 0.8

We next moved to Resin-F. The Young's modulus recorded with the samples showed a similar slight decrease with the addition of both 0.2% of L-RCDs (−8.1%) and 0.1% of RCDs (−7.2%) with respect to the pristine resin (Table 3, entries 4–6), whereas the elongation at break again went in the direction of a positive impact using coated carbon nanodots; indeed addition of the L-RCDs affected the results in a negligible way with 0.2% of L-RCDs (−3.5%), with respect to pristine Resin-F, *versus* a −16.9% observed using only 0.1% of RCDs (Table 3, entries 4–6).

In general, the obtained results showed, as expected, that the esterification of carbon dots increased their affinity for the apolar resins resulting in more homogeneous resins with a slight effect on the mechanical properties of the printed material.

Finally, some investigations on the photostability of the printed objects were performed. Among other excellent properties, carbon dots are known for their photostability, which allows them to be employed for various optical applications, including sensors, bioimaging probes and photoelectrocatalysts.^6,11^ Therefore, we envisaged high photostability even from the printed objects. In fact, for instance, on irradiating the printed Resin-R containing 0.2% of L-RCDs at 405 nm in a curing oven, we observed a reduction of the emission intensity of about 5% after an irradiation time corresponding to around 300 days of exposure to the worldwide average UV daylight dose^39^ (Fig. S16†), demonstrating the good photostability of the printed objects.

## Conclusions

In conclusion, we reported a new synthesis of red-emitting carbon dots starting from renewable materials and their long-chain surface-modification *via* an easy esterification reaction; we demonstrated, for the first time, the possibility to employ such nanoparticles as resin additives in 3D-printing vat photopolymerization and their ability to successfully transfer their fluorescence properties to the printed objects. The addition of L-RCDs to resins with various mechanical properties provides the feasibility of printing objects with different structural characteristics leading to a wide range of possible applications.

We strongly believe that these preliminary results and the interesting trend that emerged, in particular for elongation at break, could provide stimulating prospects for the world of biomedical implants.

These initial findings call for larger investigation in terms of coating, type of resin and more and may certainly enlarge the field of functionalized 3D-printed materials.

Studies are also in progress in our labs to scale up the carbon dot synthesis and to modify the functionalization to increase the amount of luminescent material in the final objects.

## Data availability

The data supporting this article have been included as part of the ESI.†

## Author contributions

L. S. supervised and coordinated the project. L. S. and M. C. F. were responsible for funding acquisition. S. M. and M. B. synthesized the compounds. S. M., E. L. and A. B. were responsible for their structural characterization. The original draft was prepared by L. S.; all the authors reviewed, edited, and approved the final version of the manuscript.

## Conflicts of interest

There are no conflicts to declare.

## Supplementary Material

NA-OLF-D4NA00617H-s001
