# Supplementary material for: Long-chain surface-modified red-emitting carbon dots as fluorescent additives for 3D printing vat-photopolymerization
Source: Nanoscale Adv. 2024 Nov 26;7(2):448–55. doi: 10.1039/d4na00617h (PMC11615732; doi:10.1039/d4na00617h)
Supplement: NA-007-D4NA00617H-s001 [file NA-007-D4NA00617H-s001.pdf]

Supplementary Information (SI) for Nanoscale Advances.
This journal is © The Royal Society of Chemistry 2024

Supporting Information

Long-chain surface-modified red-emitting carbon dots as fluorescent additives for 3D printing vat-photopolymerization

Simone Maturi,[a] Andrea Baschieri,[b] Erica Locatelli,[a] Martina Buccioli,[a] Mauro Comes Franchini[a] and Letizia Sambri* [a]

a) Department of Industrial Chemistry "Toso Montanari", University of Bologna, Via Piero Gobetti 85, 40129 Bologna, Italy.

b) Institute for Organic Synthesis and Photoreactivity (ISOF), National Research Council of Italy (CNR), Via Piero Gobetti 101, 40129 Bologna, Italy

Index

Pag.

ELECTRON MICROSCOPY-TEM ANALYSIS....2

EMISSION SPECTRA OF RCDs IN WATER SOLUTION....2

ATR-FTIR SPECTRA....3

REFLECTANCE SPECTRA OF PRINTED SAMPLES AND RCDs AND L-RCDs....4

EMISSION AND EXCITATION SPECTRA OF PRINTED SAMPLES....5

TENSILE TESTS RESULTS....9

PHOTOSTABILITY RESULTS....9

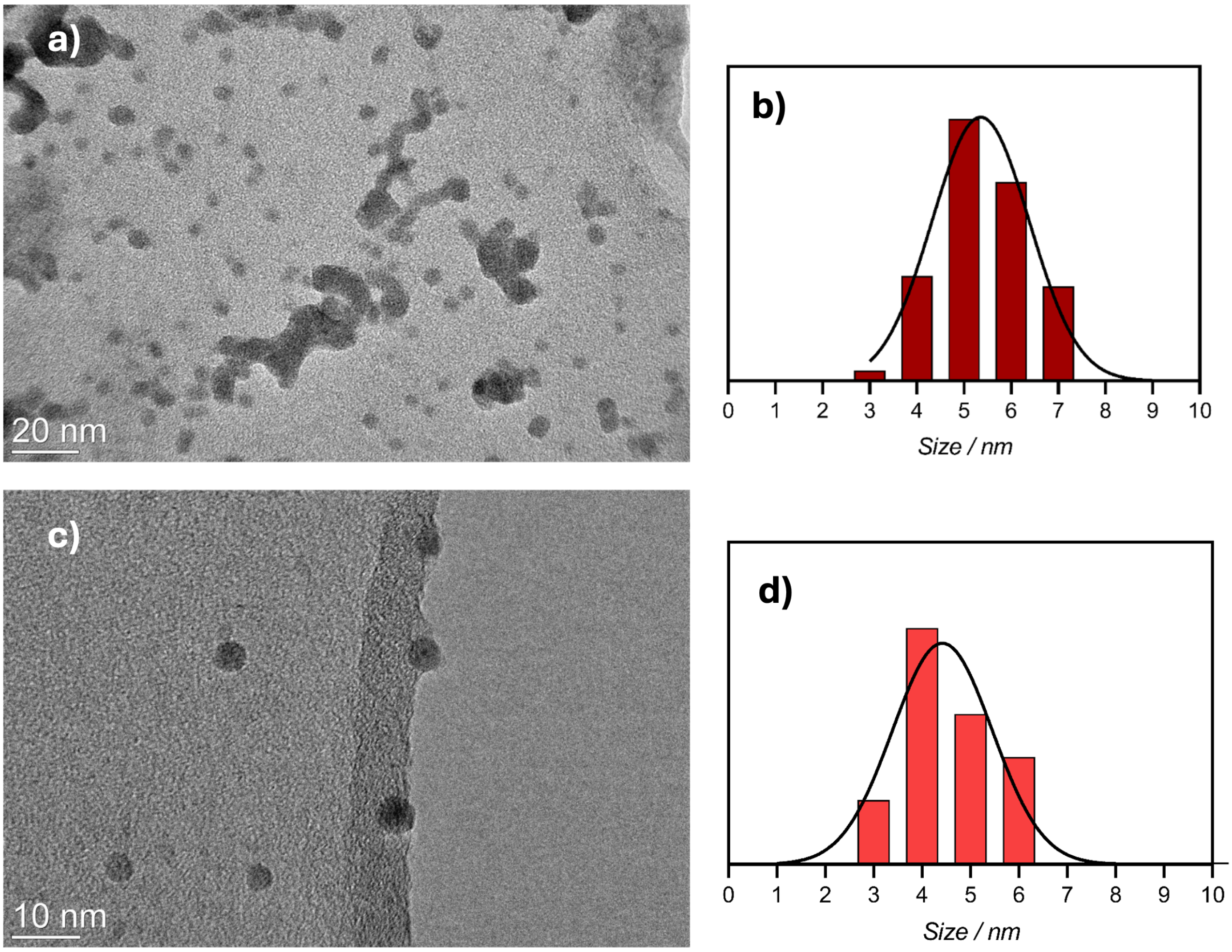

Fig. S1: A TEM image (a) of L-RCDs and their size distribution (b) showing an average diameter of 5.35 nm (SD=1.03); a TEM image of RCDs (c) and their size distribution (d) showing an average diameter of 4.42 nm (SD=1.03).

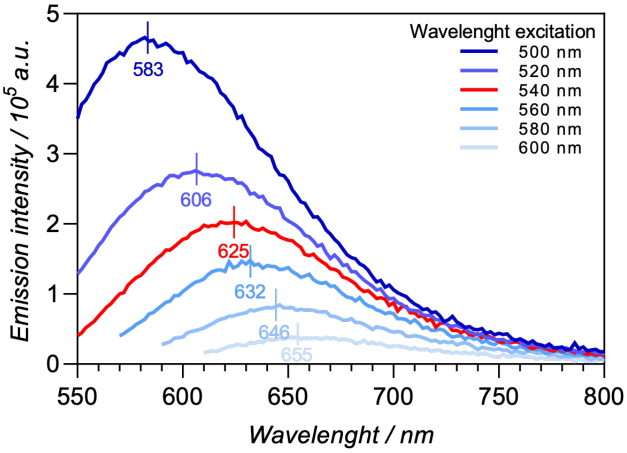

Fig. S2: Screening of the emission of **RCDs** in H_2O solution exciting in the range 500-600 nm.

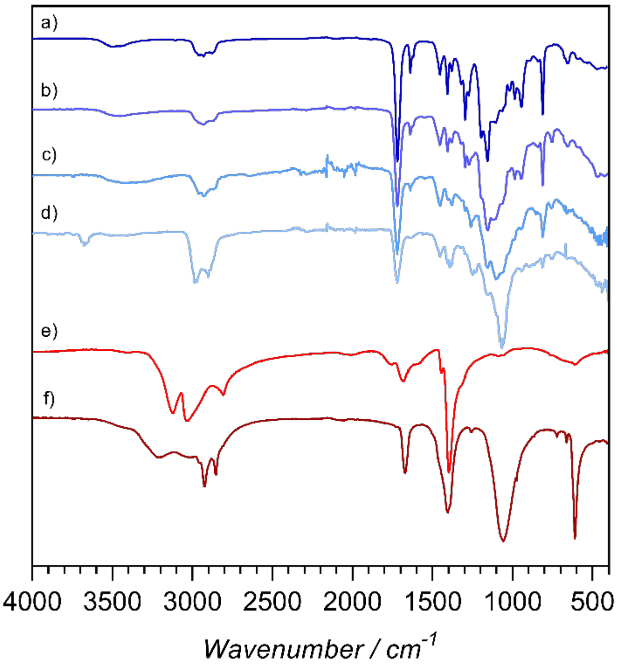

Fig. S3: ATR-FTIR spectra of the photocurable **Resin-R** before (a), after (b) the 3D printing process, printed **Resin-R** with the addition of 0.1% w/w of RCDs (c) and printed **Resin-R** with the addition of 0.1% w/w of L-RCDs (d). FT-IR spectra of the water solutions of RCDs (e) and L-RCDs (f) were added for comparison.

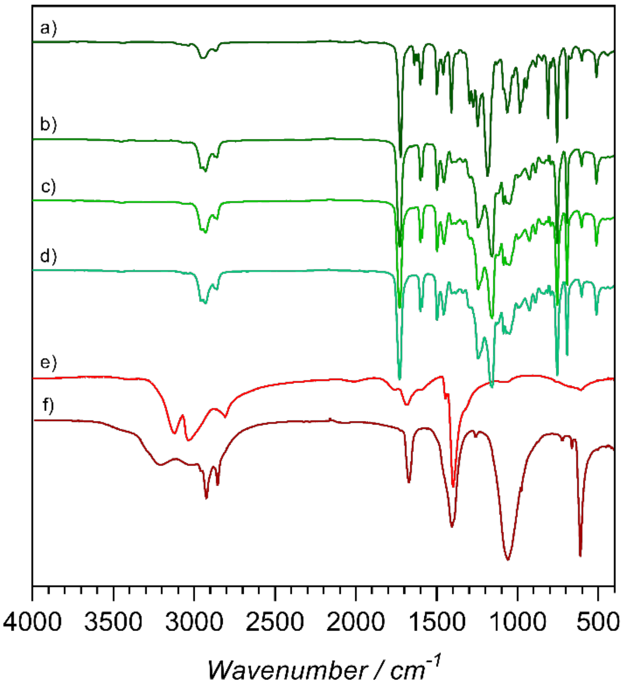

Fig. S4: ATR-FTIR spectra of the photocurable **Resin-F** before (a), after (b) the 3D printing process, printed **Resin-F** with the addition of 0.1% w/w of RCDs (c) and printed **Resin-F** with the addition of 0.1% w/w of L-RCDs (d). FT-IR spectra of the water solutions of RCDs (e) and L-RCDs (f) were added for comparison.

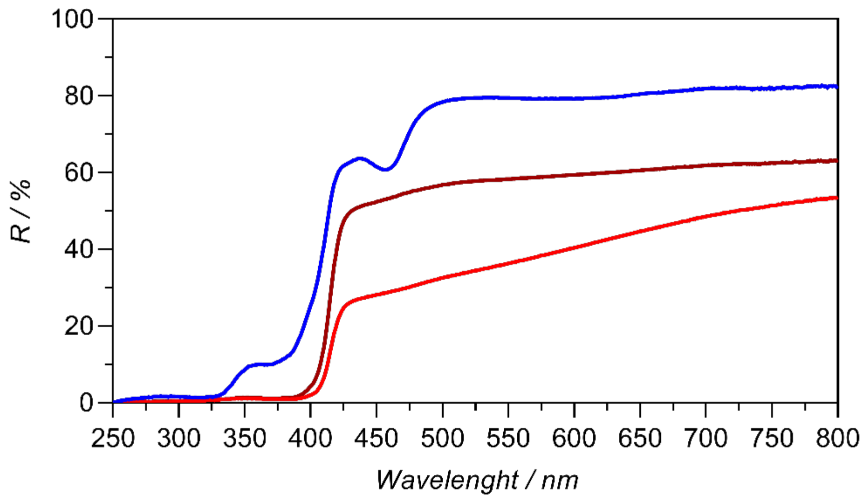

Fig. S5: Reflectance spectra of the pristine **Resin-R** (blue), **Resin-R**@L-RCDs-0.1% w/w (brown) and **Resin-R**@RCDs-0.1 % w/w (red).

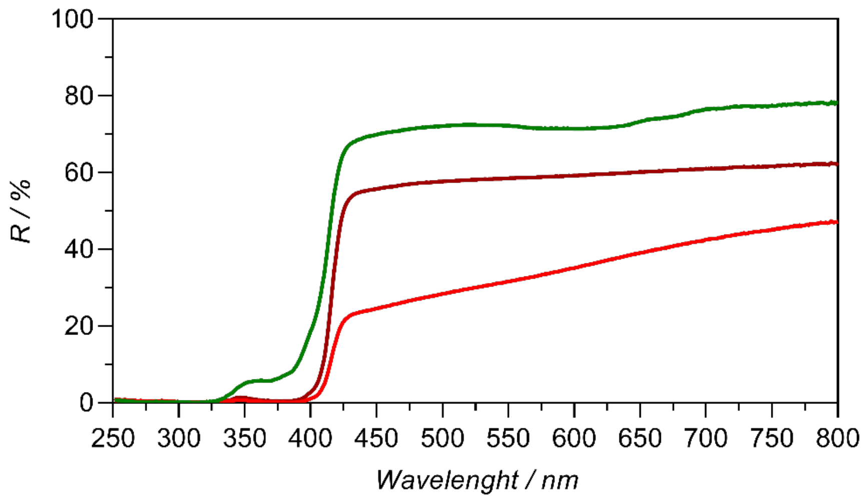

Fig. S6: Reflectance spectra of the pristine **Resin-F** (green), **Resin-F**@L-RCDs-0.1% w/w (brown) and **Resin-F**@RCDs-0.1 % w/w (red).

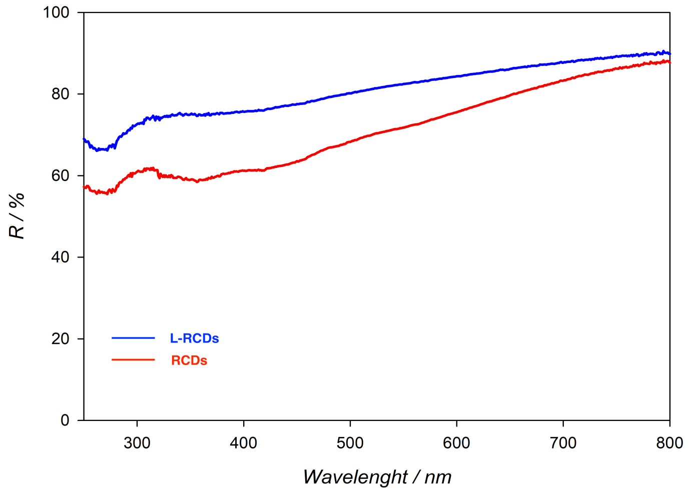

Fig. S7: Reflectance spectra of **L-RCDs** (blu) and **RCDs** (red).

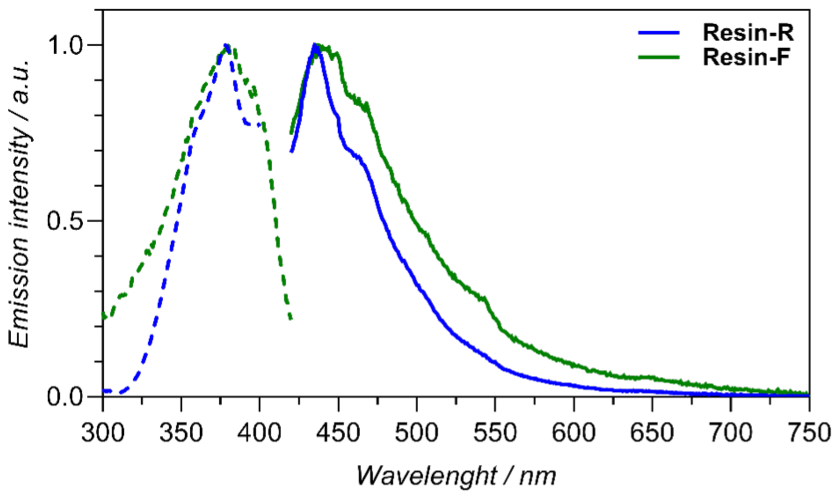

Fig. S8: Normalized excitation (dashed) and emission (solid) spectra of pristine printed **Resin-R** (blue) and **Resin-F** (green).

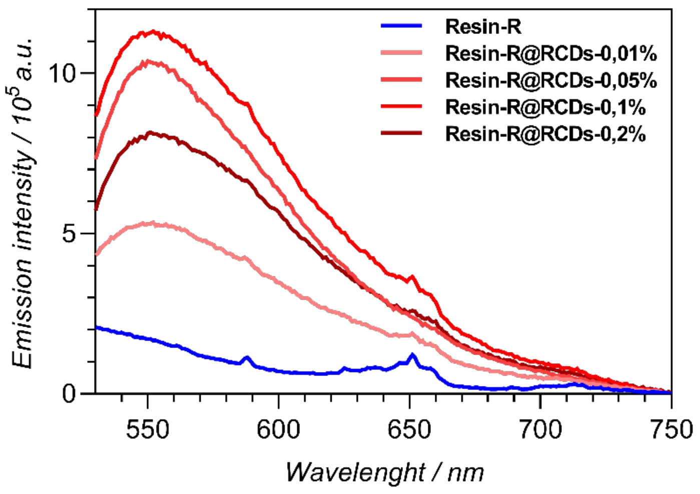

Fig. S9: Screening of the emission of **Resin-R**@RCDs in 0.01, 0.05, 0.1 and 0.2 % w/w (λ_{exc}=500 nm).

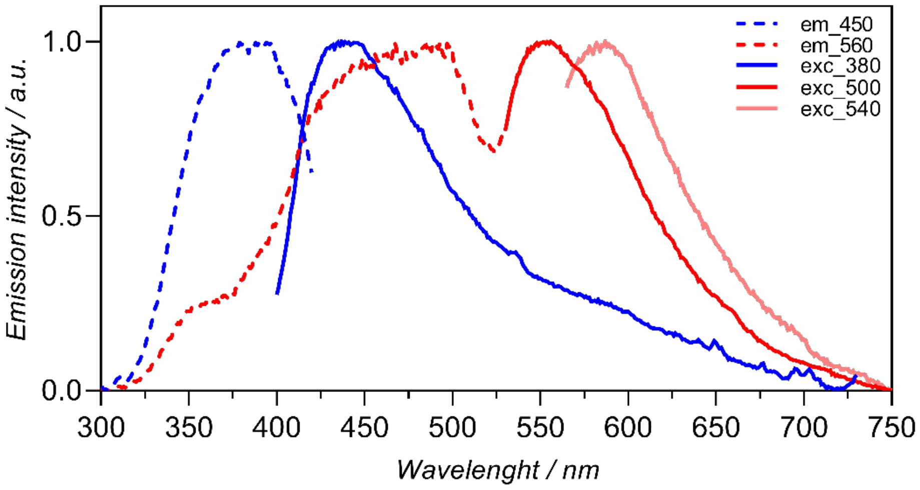

Fig. S10: Normalized excitation (dashed) and emission (solid) spectra of **Resin-R**@RCDs-0.1% w/w at different wavelengths.

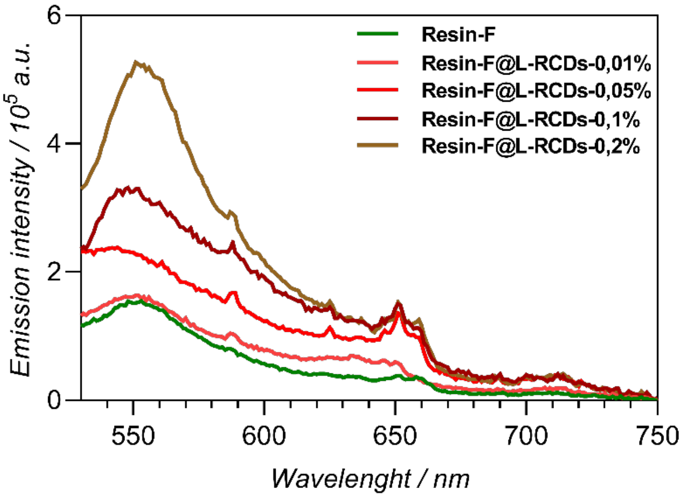

Fig. S11: Screening of the emission of **Resin-F**@L-RCDs in 0.01, 0.05, 0.1 and 0.2 % w/w (λ_{exc}=500 nm).

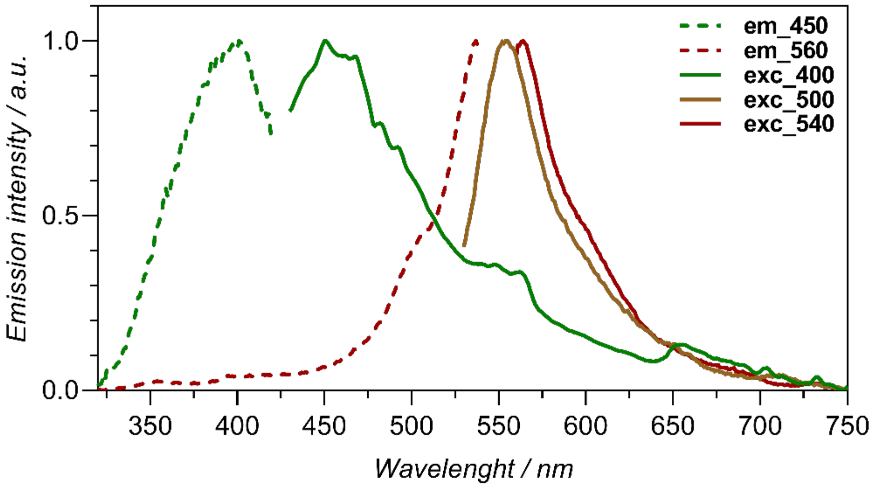

Fig. S12: Normalized excitation (dashed) and emission (solid) spectra of **Resin-F**@L-RCDs-0.2% at different wavelengths.

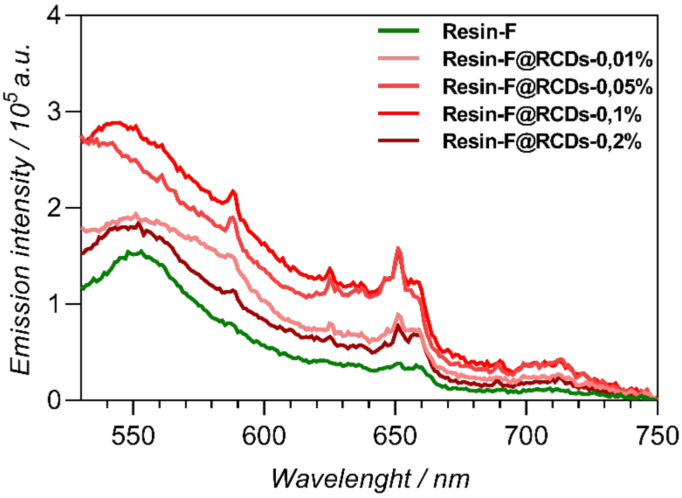

Fig. S13: Screening of the emission of **Resin-F**@RCDs in 0.01, 0.05, 0.1 and 0.2 % w/w (λ_{exc}=500 nm).

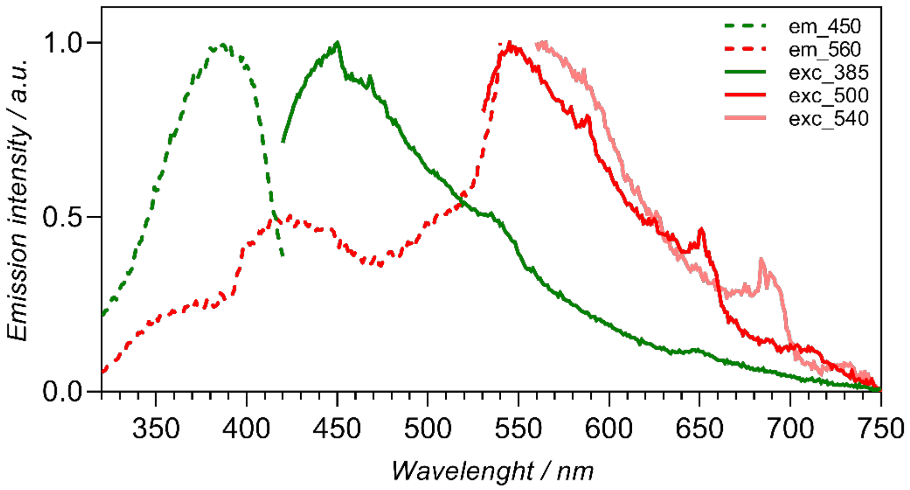

Fig. S14: Normalized excitation (dashed) and emission (solid) spectra of **Resin-F**@RCDs-0.1% w/w at different wavelengths.

Resin_R									
	0%	0,01% RCDs	0,05% RCDs	0,1% RCDs	0,2% RCDs	0,01% L-RCDs	0,05% L-RCDs	0,1% L-RCDs	0,2% L-RCDs
**Young's Modulus / MPa**	1027±32	822±95	988±47	894±41	895±38	818±30	850±74	1092±65	954±56
**Elongation at break / %**	4.2±0,7	4±1	2,3±0,9	2,2±0,5	3±1	3±1	2,5±0,6	2,2±0,2	3,9±0,4
**Tensile strenght / MPa**	31±3	30±5	19±2	17±3	24±7	27±8	19±4	22±1	31±4

Resin_F									
	0%	0,01% RCDs	0,05% RCDs	0,1% RCDs	0,2% RCDs	0,01% L-RCDs	0,05% L-RCDs	0,1% L-RCDs	0,2% L-RCDs
**Young's Modulus /MPa**	12,4±0,7	12,8±0,2	10±1	11.5±0,3	13,0±0,3	12,3±0,5	13±1	11,0±0,1	12±1
**Elongation at break / %**	17,1±0,8	16,3±0,6	12±2	14,2±0,8	16±1	19±1	18±1	17±1	16,5±0,8
**Tensile strenght / MPa**	2,0±0,1	2,0±0,1	1,4±0,3	1,5±0,1	2,0±0,2	2,3±0,3	2,3±0,2	1,9±0,2	2,0±0,1

Fig. S15: Tensile tests results of the printed objects.

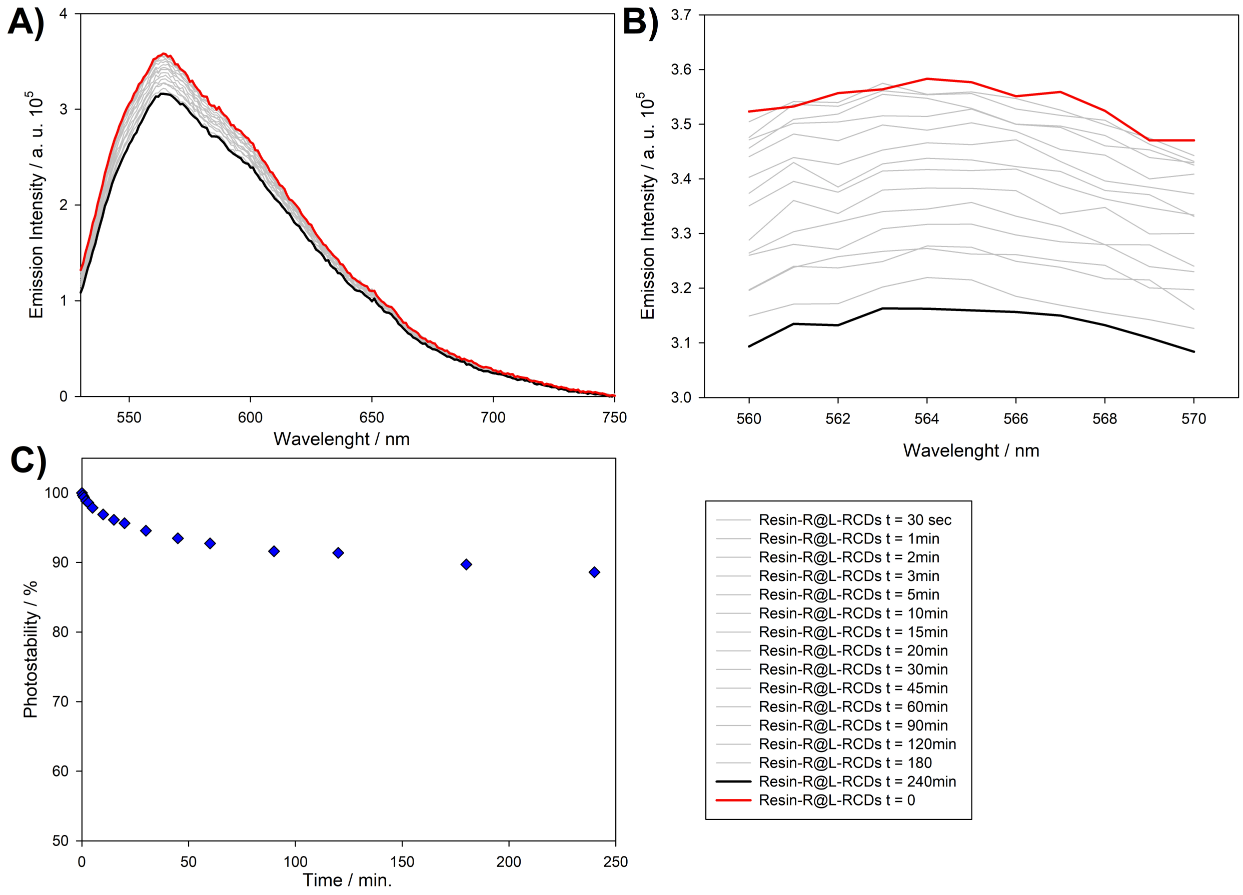

Fig. S16: Photostability test of **Resin-R** containing 0.2% of L-RCDs irradiated at 405 nm with a curing oven (LED power = 9.1 W).
